# Beneficial Effects of Vitamin D Treatment in an Obese Mouse Model of Non-Alcoholic Steatohepatitis

**DOI:** 10.3390/nu11010077

**Published:** 2019-01-03

**Authors:** Daniel Jahn, Donata Dorbath, Stefan Kircher, Anika Nier, Ina Bergheim, Kaatje Lenaerts, Heike M. Hermanns, Andreas Geier

**Affiliations:** 1 Division of Hepatology, University Hospital Würzburg, 97080 Würzburg, Germany; jahn_d@ukw.de (D.J.); dorbath_d@ukw.de (D.D.); Hermanns_H@ukw.de (H.M.H.); 2 Institute of Pathology, University of Würzburg, 97080 Würzburg, Germany; stefan.kircher@uni-wuerzburg.de; 3 Department of Nutritional Sciences, Molecular Nutritional Science, University of Vienna, 1090 Vienna, Austria; anikan88@univie.ac.at (A.N.); ina.bergheim@univie.ac.at (I.B.); 4 NUTRIM School for Nutrition and Translational Research in Metabolism, Department of Surgery, Maastricht University Medical Centre, 6200 MD Maastricht, The Netherlands; kaatje.lenaerts@maastrichtuniversity.nl; 5 Division of Gastroenterology and Hepatology, University Hospital Zürich, 8091 Zürich, Switzerland

**Keywords:** vitamin D, obesity, NAFLD, NASH, inflammation, intestine, gut–liver axis

## Abstract

Serum vitamin D levels negatively correlate with obesity and associated disorders such as non-alcoholic steatohepatitis (NASH). However, the mechanisms linking low vitamin D (VD) status to disease progression are not completely understood. In this study, we analyzed the effect of VD treatment on NASH in mice. C57BL6/J mice were fed a high-fat/high-sugar diet (HFSD) containing low amounts of VD for 16 weeks to induce obesity, NASH and liver fibrosis. The effects of preventive and interventional VD treatment were studied on the level of liver histology and hepatic/intestinal gene expression. Interestingly, preventive and to a lesser extent also interventional VD treatment resulted in improvements of liver histology. This included a significant decrease of steatosis, a trend towards lower non-alcoholic fatty liver disease (NAFLD) activity score and a slight non-significant decrease of fibrosis in the preventive treatment group. In line with these changes, preventive VD treatment reduced the hepatic expression of lipogenic, inflammatory and pro-fibrotic genes. Notably, these beneficial effects occurred in conjunction with a reduction of intestinal inflammation. Together, our observations suggest that timely initiation of VD supplementation (preventive vs. interventional) is a critical determinant of treatment outcome in NASH. In the applied animal model, the improvements of liver histology occurred in conjunction with reduced inflammation in the gut, suggesting a potential relevance of vitamin D as a therapeutic agent acting on the gut–liver axis.

## 1. Introduction

Non-alcoholic fatty liver disease (NAFLD) affects up to one-third of the Western world population, where it has become an important public health problem. Representing the hepatic organ manifestation of the metabolic syndrome, NAFLD is closely associated with obesity, hyperlipidemia and insulin resistance. Histologically, NAFLD is defined by a spectrum of lesions starting with bland liver steatosis without inflammation or fibrosis (i.e., NAFL). While liver steatosis per se is a rather benign state, it can progress to non-alcoholic steatohepatitis (NASH) which involves hepatic inflammatory changes and apoptosis leading to organ injury and fibrosis, and can ultimately advance to end-stage liver disease including cirrhosis and cancer [1,2]. Due to the continuously increasing global burden of obesity, NAFLD is currently among the most common causes of liver transplantation and is projected to become an even larger problem in the near future [3]. Since no pharmacological therapy has been approved for the treatment of NAFLD so far, there is an urgent necessity to define pathophysiology-based strategies of future interventions [4].

Vitamin D is an endocrine signaling molecule playing a major role in human health. To maintain adequate physiological levels, vitamin D can be produced in the skin from a 7-dehydrocholesterol precursor upon UV-B radiation or can be taken up with the diet. However, due to modern life-style and the fact that only few foods contain high amounts of vitamin D, many people have vitamin D levels that are below the recommended range. Especially in the obese population, vitamin D deficiency (commonly defined as circulating 25-OH vitamin D levels of 30 ng/mL or less) is a very common phenomenon [5,6,7]. In fact, a close correlation between body mass index (BMI) and 25-OH vitamin D level has been described with −1.3 nmol/L vitamin D per 1 kg/m^2^ increase in BMI [8]. Importantly, vitamin D deficiency is associated with certain metabolic complications and with the progression of NAFLD. For example, low vitamin D levels are correlated with several markers of insulin resistance such as plasma glucose, insulin, HOMA-IR and adiponectin in non-diabetic adults [9,10]. In individuals with NAFLD, decreased 25-OH vitamin D levels can be found in close association to the histological severity of hepatic steatosis, necro-inflammation and fibrosis [11]. Moreover, vitamin D deficiency (defined as <20 ng/mL in this study) increases the likelihood of definitive NASH in US adult patients with NAFLD [12]. Despite the wealth of these association studies, it is presently difficult to identify a functional link between low serum vitamin D and disease progression as a causal relationship.

From a physiological point of view, the vitamin D that is produced in the skin or taken up from the diet (i.e., vitamin D3/cholecalciferol) is undergoing 25-hydroxylation in the liver leading to 25-OH vitamin D3 (calcidiol). 25-OH vitamin D represents the major form of the vitamin in the circulation. Subsequently, 25-OH vitamin D is undergoing 1-hydroxylation in the kidney to be converted into its biologically active form 1,25-dihydroxyvitamin D3 (i.e., calcitriol). Calcitriol is an endocrine signaling molecule that binds to the vitamin D receptor (VDR, NR1I1), which serves as a ligand-activated transcription factor to control the expression of a large spectrum of diverse target genes in various tissues [13,14]. As one of the classical endocrine functions, calcitriol/VDR controls the uptake of dietary calcium in the gut [15]. Besides that, more recent studies suggest that the activation of 25-OH vitamin D to 1,25-dihydroxyvitamin D can also be carried out by certain immune cell types, implicating a role of active vitamin D and VDR as local immuno-modulators [16].

In fact, a number of beneficial effects of vitamin D on metabolic organs including adipose tissue and liver have been described in the context of obesity and chronic inflammatory disorders [17]. For example, vitamin D substitution in obese or insulin-resistant subjects leads to the amelioration of insulin resistance without affecting insulin secretion [18,19]. Similarly, a therapeutic potential of vitamin D has been suggested in NAFLD but the precise effects of high-dose vitamin D intervention (beyond substitution) versus untreated 25-OH vitamin D deficiency has not been studied in detail. The aim of the present study was to investigate the preventive and interventional effects of high-dose vitamin D treatment in a dietary mouse model of obesity and vitamin D deficiency resembling the human hepatic phenotype of fibrosing NASH.

## 2. Material and Methods

### 2.1. Animals and Diets

**Figure 1:**
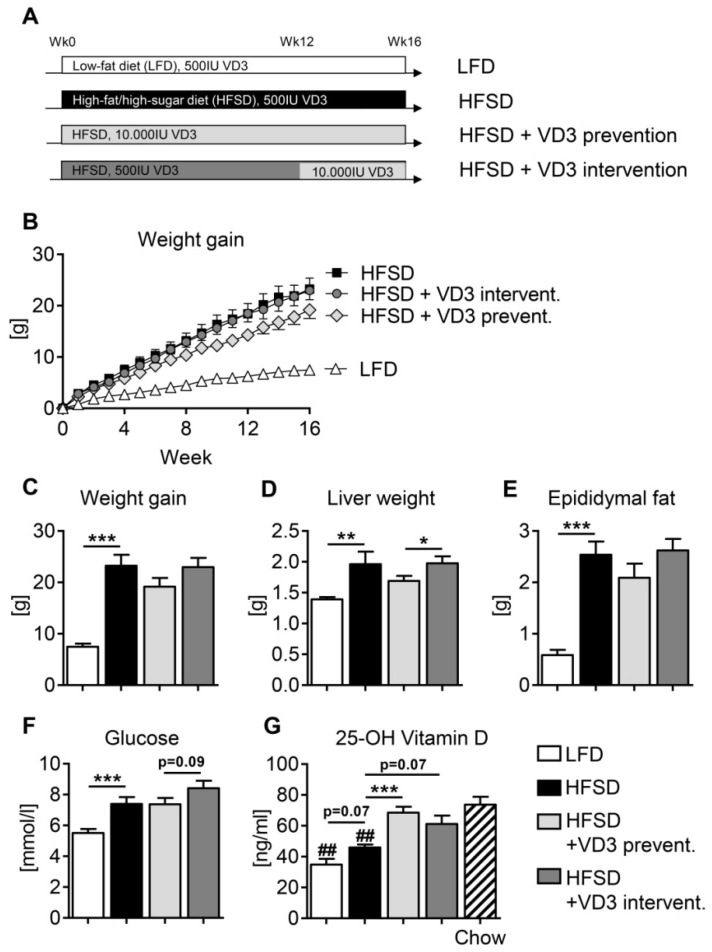
Effects of vitamin D treatment on general metabolic parameters in HFSD-fed mice. (**A**) Experimental set-up. Male C57BL/6J mice received high-fat/high-sugar diet (HFSD) or low-fat control diet (LFD) for 16 weeks. Vitamin D3 (VD3) treatment was either performed from week 0 (HFSD + VD3 prevention) or week 12 (HFSD + VD3 intervention) onwards (see Material and Methods for further details). At the end of the study, general metabolic parameters were measured: (**B**,**C**) weight gain, (**D**) liver weight, (**E**) epididymal fat mass, (**F**) fasting blood glucose and (**G**) serum 25-OH vitamin D levels. Data in (**B**–**F**) represent mean ± SEM derived from *n* = 9 for LFD and *n* = 10 for all the other groups. Data in (**G**) represent mean ± SEM derived from *n* = 6 for LFD and *n* = 7 for all other groups. ## in (**G**) indicate *P* < 0.01 compared to chow-fed mice.

Mice were housed in groups of 5 animals on a 12 h light/dark schedule and had free access to food and drinking water. Eight-week-old male C57BL/6J mice (Charles River, Sulzfeld, Germany) were used for the study and were assigned to one of the following groups: “LFD” mice received a low-fat control diet (LFD, E15051, Ssniff Spezialdiäten GmbH, Soest, Germany) containing 500 IU vitamin D3 per kg diet for 16 weeks. The “HFSD” group received a high-fat/high-sugar Surwit diet (59 kcal% from fat, E15772, Ssniff Spezialdiäten GmbH, Soest, Germany) containing 500 U vitamin D3 per kg diet for 16 weeks. As this vitamin D3 content of 500 IU per kg diet is only half of the amount recommended by the National Research Council [20], this feeding regimen should cause a state of moderate vitamin D deficiency. In the “HFSD + VD3 prevention” and “HFSD + VD3 intervention” groups, the vitamin D3 content of the HFSD was modified as follows. In the “HFSD + VD3 prevention” group, animals were fed with HFSD containing a high dose of 10,000 IU vitamin D3 per kg diet for the entire duration of the study (16 weeks). In contrast, the “HFSD + VD3 intervention” group received HFSD diet with 500 IU vitamin D3 for 12 weeks and then switched to the HFSD containing 10,000 IU vitamin D3 for further 4 weeks (see experimental set-up in Figure 1A). Additionally, the drinking water of all three HFSD groups was enriched with sucrose (18.9 g/L) and fructose (23.1 g/L). This dietary treatment (HFSD combined with sucrose/fructose-enriched drinking water) has previously been shown to induce NASH and fibrosis in mice after 16 weeks of feeding [21]. At the end of the study, samples were collected after 8–12 h of fasting. All animal experiments were performed with the approval of the local authorities (Regierung von Unterfranken, Würzburg, Germany).

### 2.2. Serum 25-OH Vitamin D Levels

Serum vitamin D levels were determined with the 25-OH Vitamin D ELISA (REA300/96, BioVendor, Kassel, Germany) according to the manufacturer`s instructions. In addition to the four experimental groups mentioned in the previous section, age-matched male C57BL/6J mice that were fed with a standard lab chow (supplemented with 1000 IU vitamin D3/kg diet) were included in the analysis in order to obtain normal vitamin D reference values.

### 2.3. Histological Analyses

Formalin-fixed and paraffin-embedded liver samples were sectioned and stained with either haematoxylin and eosin (H&E) or Ladewig trichrome. Specimens were analyzed by a pathologist blinded to the experimental groups (SK). Liver steatosis, inflammation, hepatocyte ballooning and fibrosis were scored according to Kleiner et al. [22]. Using this method, individual scores for steatosis (0–3), inflammation (0–3) and ballooning (0–2) were provided and were added up to determine the NAFLD activity score (NAS) as a semi-quantitative measure of disease severity. Fibrosis was scored separately from 0 to 4 with 0 indicating absence of fibrosis and 4 indicating liver cirrhosis. Images were taken with a BZ-9000 microscope and processed with the BZ-II Analyzer software (Keyence, Neu-Isenburg, Germany).

### 2.4. RNA Isolation and Gene Expression Analysis

Total RNA from liver and ileum samples was extracted with the Nucleo Spin RNA kit (Machery-Nagel, Dueren, Germany) according to the manufacturer’s instructions. Reverse transcription was carried out with the High-Capacity cDNA Reverse Transcription Kit (Thermo Fisher Scientific, Dreieich, Germany). Gene expression was analyzed by quantitative PCR (qPCR) with the SYBR Select Master Mix on a ViiA7 (Thermo Fisher Scientific, Dreieich, Germany). Relative mRNA expression was calculated by the comparative ΔΔCT method. Normalization was done using *Rplp0* and *Vil1* as housekeeping genes for liver and intestinal samples, respectively.

### 2.5. Serum Endotoxin

Endotoxin concentrations in serum were measured as previously described [23]. In brief, a commercially available endpoint limulus amebocyte lysate assay with a concentration range of 0.015–1.2 EU/mL (Charles River, Lyon, France) was used to determine bacterial endotoxin levels.

### 2.6. Lipopolysaccharide (LPS)-Binding Protein ELISA

Mouse LPS-binding protein (LBP) was measured in serum using a commercially available mouse LBP ELISA (Hycult Biotech, Uden, The Netherlands) according to the manufacturer’s instructions. The detection limit of the assay was 0.8 ng/mL.

### 2.7. Statistical Analyses

Statistical analyses were performed using GraphPad Prism 6 (GraphPad Software Inc, San Diego, CA, USA). Data was analyzed by the Mann–Whitney test. Statistically significant differences between groups are indicated by asterisks: * *P* < 0.05, ** *P* < 0.01, and *** *P* < 0.001. Samples sizes were *n* = 9–10 per group except for the analysis serum vitamin D levels (*n* = 6–7 per group).

## 3. Results

### 3.1. Effects of Vitamin D Treatment on Metabolic Parameters and on Serum 25-OH Vitamin D Levels in HFSD-Fed Mice

As expected, HFSD mice developed obesity characterized by significantly enhanced body weight gain, increased liver weight and increased epididymal fat mass compared to LFD-fed animals (Figure 1B–E). In addition, mice in the HFSD group showed elevated fasting blood glucose levels indicative of reduced glucose tolerance and/or impaired insulin sensitivity (Figure 1F). Although neither the preventive nor the interventional vitamin D treatment could fully counteract these unbeneficial metabolic changes, a moderate but non-significant decrease of body weight, liver weight and fat mass was noted in the preventive treatment group (Figure 1C–E).

Notably, both the LFD and the HFSD control groups (receiving 500 IU of vitamin D3 per kg diet throughout the study) showed significantly decreased serum levels of 25-OH vitamin D compared to age-matched mice that were fed standard chow not depleted for vitamin D (supplemented with 1000 IU vitamin D3/kg diet) (Figure 1G). Moreover, both the preventive and the interventional high-dose vitamin D treatments in the HFSD groups increased serum 25-OH vitamin D levels compared to the vitamin D-depleted HFSD group (*P* < 0.001 and *P* = 0.07), leading to an almost complete normalization at the end of the study. These data demonstrate that the applied vitamin D-depleted HFSD-feeding model accurately resembles the metabolic state of hypovitaminosis D which is well recognized in the majority of human patients with obesity and/or NAFLD.

### 3.2. Effects of Vitamin D Treatment on NAFLD Activity and Liver Fibrosis

**Figure 2:**
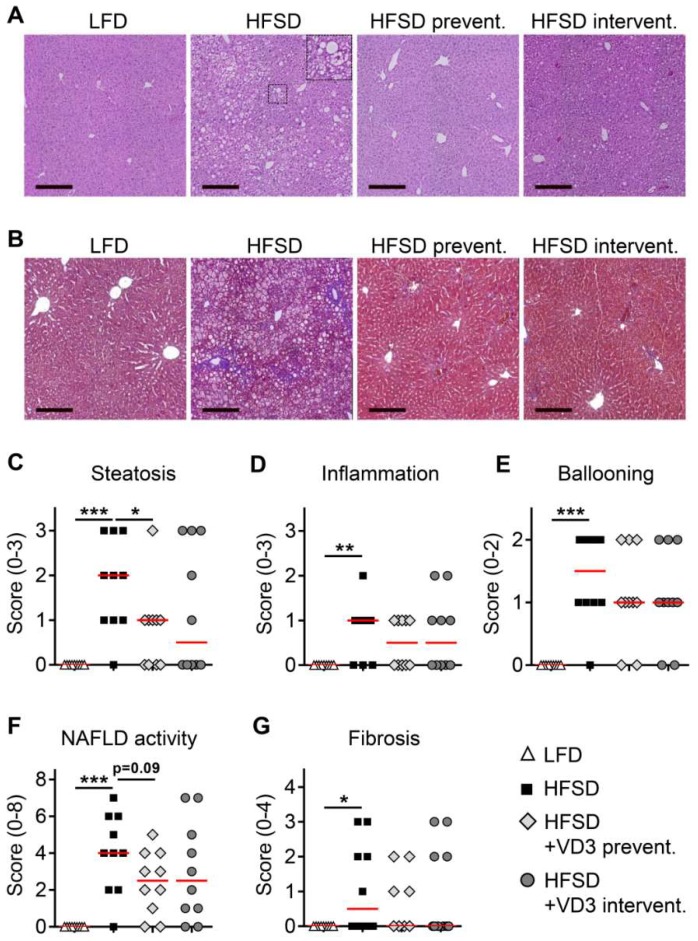
Effects of preventive and interventional vitamin D treatment on liver histology and NAFLD activity after 16 weeks of HFSD-feeding. Representative H&E-stained and trichrome-stained liver sections are shown in (**A**) and (**B**), respectively (bars, 200 µm). The enlarged picture detail in (**A**) shows an example of a ballooned hepatocyte. (**C**–**G**) Steatosis, inflammation, hepatocyte ballooning and liver fibrosis were scored according to Kleiner et al. [22]. Data are presented as scatter and dot plots with median. Sample sizes for all analyses shown in (**C**–**G**) were *n* = 9 for LFD and *n* = 10 for all the other groups.

Liver histology was analyzed in tissue sections stained with H&E (Figure 2A) or Ladewig trichrome (Figure 2B). As expected, low vitamin D-treated HFSD-feeding induced significant increases in liver steatosis, inflammation and hepatocyte ballooning compared to the LFD-fed group (Figure 2C–E), resulting in the presence of definite NASH in the majority of these animals (median NAFLD activity score of 4; Figure 2F). In addition, liver fibrosis was present in 50% of the low vitamin D-treated HFSD-fed mice, which is in line with a previous report using a similar experimental set-up based on HFSD-feeding combined with sucrose/fructose-enriched drinking water [21].

With regard to the therapeutic effects of vitamin D, preventive treatment with 10,000 IU per kg diet caused a significant decrease of steatosis (Figure 2C) and slight but non-significant improvements of inflammation and ballooning (Figure 2D,E). Importantly, this resulted in a clear trend towards lowered overall NAFLD activity (Figure 2F, *P* = 0.09). In addition, a slight non-significant decrease of liver fibrosis was observed in the preventive treatment group (Figure 2G). Similar beneficial effects on liver histology could also be found after interventional vitamin D treatment, however, these effects were less pronounced and did not reach significance. This indicates that timely onset of vitamin D treatment is critical for a relevant improvement of NASH in the applied animal model.

### 3.3. Vitamin D Treatment Reduces the Expression of Lipogenic, Inflammatory and Pro-Fibrotic Genes in the Liver of HFSD-Fed Mice

**Figure 3:**
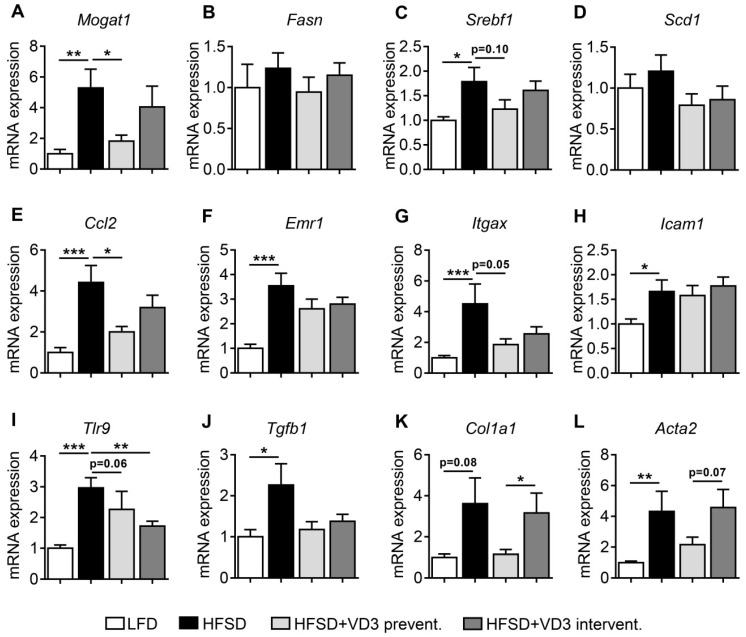
Effects of vitamin D treatment on hepatic gene expression after 16 weeks of HFSD-feeding. Expression levels of lipogenic (**A**–**D**), inflammatory (**E**–**I**) and pro-fibrotic (**J**–**L**) genes were analyzed by qPCR. Data represent mean ± SEM derived from *n* = 9 for LFD and *n* = 10 for all the other groups. Relative gene expression was normalized to *Rplp0* mRNA levels.

In accordance with the induction of obesity and histologically defined NASH in the applied animal model, the expression of key genes involved in lipogenesis (Figure 3A–D), inflammation (Figure 3E–I), and fibrogenesis (Figure 3J–L) was increased in low vitamin D-treated HFSD-fed mice compared to LFD controls. In line with the improvements of liver histology shown in Figure 2, the preventive vitamin D treatment group showed significant reductions of the expression levels of certain disease marker genes. These genes included *Mogat1* which encodes the lipogenic enzyme monoacylglycerol O-acyltransferase 1 (MGAT1) and *Ccl2* encoding the pro-inflammatory chemokine monocyte chemoattractant protein 1 (MCP-1). In addition, the expression of other factors involved in lipogenesis (*Srebf1* encoding sterol regulatory element-binding protein 1) and inflammation (*Itgax* encoding the cell-surface protein Cd11c and *Tlr9* encoding Toll-like receptor 9) tended to be lower (*P* ≤ 0.1) in the preventive treatment group, although these effects did not reach statistical significance. In comparison to the preventive treatment arm, the effects of the interventional treatment with high-dose vitamin D were less pronounced for most of the analyzed genes without statistical significance compared to the low vitamin D-treated HFSD-fed group (with the exception of *Tlr9*).

### 3.4. Effects of Vitamin D on the Intestinal Expression of Fgf15 and Hepatic Genes Involved in Bile Acid Synthesis

**Figure 4:**
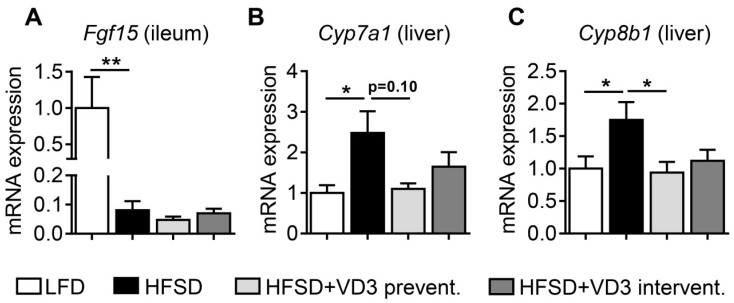
Expression of intestinal *Fgf15* and hepatic genes involved in bile acid synthesis in vitamin D-treated mice after 16 weeks of HFSD-feeding. (**A**) Expression of *Fgf15* in the ileum and (**B**,**C**) *Cyp7a1* and *Cyp8b1* expression in the liver was analyzed by qPCR. Data represent mean ± SEM derived from *n* = 9 for LFD and *n* = 10 for all the other groups. Relative mRNA expression was normalized to *Vil1* (ileum) or *Rplp0* (liver) transcript levels.

We aimed to obtain insights into the mechanisms that could underlie the beneficial effects of high-dose vitamin D on HFSD-induced obesity, liver histology and hepatic gene expression. Fibroblast growth factor 15 (FGF15) is an intestine-derived hormone that regulates bile acid (BA) synthesis in the liver by down-regulation of the rate-limiting enzyme CYP7A1 [24,25]. In addition to this, FGF19 (the human ortholog of FGF15) has been suggested to improve adiposity and liver steatosis in obese mouse models [26,27] and the mouse *Fgf15* gene has been defined as a direct transcriptional target of VDR [28]. Interestingly, decreased levels of FGF19 have been reported in patients with NAFLD and other metabolic disorders, however, the underlying mechanisms are poorly defined [29]. Therefore, we investigated the expression of intestinal *Fgf15* to analyze its potential contribution to the beneficial effects of vitamin D described above. In fact, we observed a marked decrease of *Fgf15* mRNA levels in the ileum of HFSD-fed animals compared to LFD controls (Figure 4A). However, neither preventive nor interventional treatment of HFSD-fed mice with high-dose vitamin D could restore ileal *Fgf15* expression in this experimental set-up. This suggests that the intestinal VDR-FGF15 pathway is not the primary metabolic mediator of the beneficial effects of vitamin D in the applied NASH mouse model.

Besides alterations in serum FGF19 levels, human NAFLD is characterized by marked changes of BA metabolism which have been suggested to contribute to disease activity by various mechanisms [30,31,32]. Based on this, we analyzed the expression of genes involved in BA biosynthesis in the livers of HFSD-fed mice. In line with a dysregulation of BA metabolism in NAFLD, mRNA levels of *Cyp7a1* and *Cyp8b1* were increased in HFSD-fed mice on low vitamin D compared to LFD controls (Figure 4B,C). Interestingly, these inductions of *Cyp7a1* and *Cyp8b1* were completely (*Cyp8b1*) or partially (*Cyp7a1*; *P* = 0.1 compared to HFSD) abrogated by preventive vitamin D treatment. This suggests that one mechanism underlying the beneficial effects of vitamin D in our NASH model is based on normalization of BA metabolism. Since these effects occurred in the absence of changes in intestinal *Fgf15,* the repression of BA synthesis enzymes by vitamin D likely reflects a direct effect in the liver. In line with this notion, Han and Chiang previously demonstrated that ligand-bound VDR inhibits *CYP7A1* expression in human hepatocytes by a direct transcriptional mechanism [33].

### 3.5. Vitamin D Attenuates the HFSD-Induced Up-Regulation of Inflammatory Markers in the Intestine

Obesity and metabolic disorders are generally associated with a chronic low-grade inflammation in the gut and a partial impairment of the intestinal barrier, e.g., by down-regulation of tight junction proteins, leading to increased intestinal permeability [34,35,36]. Increased permeability of the gut (also termed “leaky gut”) can, in turn, lead to the translocation of bacterial metabolites such as LPS into portal blood and the systemic circulation. By this mechanism, intestinal inflammation and impaired intestinal barrier function can promote NASH and liver fibrogenesis in animal models and human patients [37,38,39,40]. Based on this, serum levels of LPS and the endotoxemia surrogate marker LBP were measured in blood samples of mice. However, neither LPS nor LBP was significantly increased in HFSD-fed mice on low vitamin D as compared to lean LFD controls (Figure S1). This suggests that increased translocation of LPS from the gut to the liver may not be the primary driving force of liver inflammation in the applied experimental set-up.

**Figure 5:**
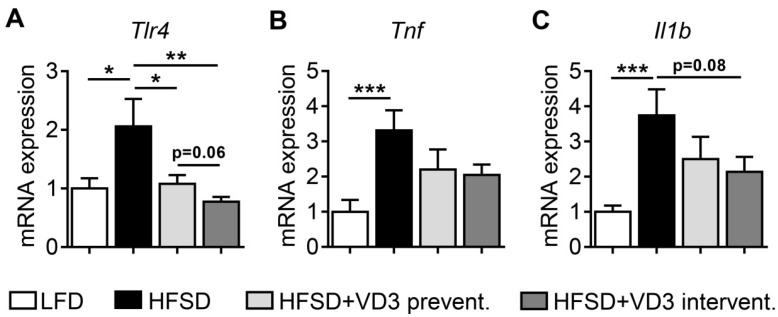
Intestinal expression of inflammatory marker genes in vitamin D-treated mice after 16 weeks of HFSD-feeding. (**A–C**) Gene expression of *Tlr4*, *Tnf* and *Il1b* was measured by qPCR after reverse transcription of total RNA extracted from the ileum. Data represent mean ± SEM derived from *n* = 9 for LFD and *n* = 10 for all the other groups. Relative mRNA expression was normalized to *Vil1* transcript levels.

The expression of pro-inflammatory marker genes in the ileum of HFSD-fed mice was, however, significantly changed. In line with previous studies, pro-inflammatory genes including *Tlr4, Tnf* and *Il1b* were up-regulated in the ileum of low vitamin D-treated HFSD-fed animals in comparison to LFD controls (Figure 5). Interestingly, this HFSD-induced intestinal inflammation was ameliorated by vitamin D. This became evident by a complete normalization to LFD control levels for *Tlr4* expression with both high-dose vitamin D prevention and intervention. In addition, *Tnf* and *Il1b* expression levels clearly decreased in both vitamin D treatment groups without statistical significance. These observations indicate that, in addition to the hepatic effects on BA homeostasis, vitamin D has immunomodulatory effects in the gut that may contribute to the observed improvements of NASH by reducing obesity-associated intestinal inflammation. The absence of increases in LPS or LBP serum levels suggests that these inflammatory changes may not be necessarily accompanied by an impairment of intestinal barrier function in our experimental model. As one possible explanation for this finding, intestinally derived cytokines such as TNF of IL-1 may act as endocrine factors directly on hepatic cells via the portal circulation to promote liver inflammation and fibrogenesis in NASH. However, this aspect will need further validation in future studies.

## 4. Discussion

The present study addresses the effects of preventive and interventional high-dose vitamin D therapy in a dietary mouse model of obesity and vitamin D deficiency resembling fibrosing NASH in humans. Major findings of the study were: (i) Preventive but not interventional treatment with high-dose vitamin D showed a clear trend to counteract HFSD-induced body weight gain and adipose tissue expansion. (ii) Increased 25-OH vitamin D serum levels in HFSD-fed animals were associated with an improvement of liver steatosis, a trend towards reduced overall NASH activity and a slight non-significant decrease of liver fibrosis. (iii) Preventive vitamin D treatment led to a variable degree of reduction in expression levels of hepatic lipogenic genes, together with a decrease in inflammatory and pro-fibrotic targets. (iv) A similar reversal of pro-inflammatory genes could be detected in the intestine. (v) In the liver, the NAFLD-associated induction of genes involved in BA synthesis (*Cyp7a1* and *Cyp8b1*) was markedly attenuated by vitamin D.

Previous preclinical studies in rodent models are partially controversial with regard to the metabolic effects of vitamin D/VDR on obesity and NAFLD. While some studies have shown beneficial outcomes [41,42,43,44,45,46,47,48], others suggest rather undesirable metabolic features of vitamin D and its receptor, VDR, in certain animal models [49,50,51]. One explanation for the diversity of the results could be related to considerable differences in study design. These, for example, include different doses and different biological forms of vitamin D that are provided (e.g., cholecalciferol, 1,25-dihydroxyvitamin D, or pharmacological vitamin D analogues such as 1α-hydroxy-cholecalciferol or paricalcitol), as well as different routes of their administration (diet/oral vs. injection). In this regard, it was an important aspect of our study design that we used oral administration (via the diet) of vitamin D in the chemical form of cholecalciferol. This treatment regimen would correspond to the use of vitamin D as a nutritional supplement in humans, which is simple to use, cost-efficient and known to be generally well-tolerated in doses as high as 4000–10,000 IU per day in clinical practice [52,53].

Among the previously published preclinical studies reporting an improvement of NAFLD by vitamin D, Nakano and coworkers have investigated the impact of sunlight therapy on the progression of NAFLD in rats fed a choline-deficient L-amino acid-defined (CDAA) diet [41]. Comparable to low 25-OH vitamin D levels reported in human NAFLD [11], 25-OH vitamin D and 1,25-(OH)2 vitamin D serum levels decreased during CDAA feeding, however, it remained unclear whether this decrease is due to the progression of NAFLD or simply reflecting a different dietary content of the CDAA diet compared to standard chow. Consistent with our findings from the present study, Nakano et al. reported that therapeutic intervention with the prodrug 1α-hydroxy-cholecalciferol ameliorated histology as measured by a decreased hepatitis area.

Yin et al. investigated a nutritional rat model of high-fat diet (HFD) compared to a normal-fat diet group with putative average vitamin D and supplemented the high-fat diet animals with intraperitoneal injection of three different doses of 1,25-dihydroxyvitamin D3 [43]. Here, vitamin D intervention prevented HFD-induced weight gain and hepatic steatosis, however, effects on liver inflammation and fibrogenesis were not addressed in this study. Moreover, the effects of the experimental diets on serum 25-OH vitamin D levels were not reported. Whether high-fat diet per se causes hypovitaminosis D in this model remains unclear.

Another rat study by Roth et al. used a custom rodent high-fat and corn syrup (HFCS) diet with a vitamin D content that was either normal (1000 IU vitamin D) or depleted (25 IU vitamin D) [42]. In this study, experimental vitamin D deficiency exacerbated the effects of HFCS on liver steatosis and inflammation, thus resulting in increased disease activity measured by the NAFLD activity score [22]. However, significant liver fibrosis was not reported in this model and thus the effects of vitamin D deficiency on fibrogenesis could not be evaluated in this study. Similar to that, Kong et al. used high-fat diets, either devoid of vitamin D or containing a normal vitamin D content (1000 IU vitamin D) [44]. For intervention, 1,25-dihydroxyvitamin D3 at a dose of 5 ng/g body weight was applied by intramuscular injection twice a week. In this study, vitamin D deficiency caused increased NAFLD severity while treatment with 1,25-dihydroxyvitamin D3 had an opposite effect. However, the scoring system used to evaluate NASH and liver fibrosis in this study was apparently slightly different from the histopathological standards presently used in most clinical trials [54].

The studies summarized above point to a beneficial role of vitamin D (and different vitamin D analogs) on liver healthy in the context of obesity and metabolic disease. However, it is important to note that not all of these studies addressed the therapeutic effect of vitamin D on bona fide NASH as the progressive and clinically most relevant form of NAFLD. In our study, we used a mouse model that was based on feeding of an HFSD and additional consumption of sucrose/fructose-enriched drinking water. In line with a previous report [21], this led to the development of bona fide NASH (defined by the presence of liver steatosis, inflammation and ballooning) in the majority of HFSD-fed control mice as well as the occurrence of histo-pathologically detectable liver fibrosis in half of these animals. This progressive histo-pathological phenotype is an important aspect of NAFLD animal models form a clinical point of view [54], thus underlining the clinical relevance of our results.

Overall, our data and other preclinical reports point to a beneficial role of dietary vitamin D supplementation in NASH as the progressive form of NAFLD. Moreover, our data imply that timely initiation of this intervention may be crucial to prevent disease progression and point to an important role of the gut as a primary target organ of the physiological and pharmacological actions of vitamin D. A randomized placebo-controlled pilot study conducted by our group within the Swiss Association for the Study of the Liver (SASL) network has recently reported beneficial effects of vitamin D treatment on serum ALT levels in patients with histologically defined NASH [55]. Together with the present study, this warrants further investigation on the usefulness of vitamin D as a treatment option for NASH and the underlying mechanisms in future clinical and preclinical trails.

## Supplementary Materials

The following are available online at http://www.mdpi.com/2072-6643/11/1/77/s1, Figure S1: Effects of preventive and interventional vitamin D treatment on serum levels of lipopolysaccharide (LPS) and LPS-binding protein (LBP).
