# Supplementary material for: Beneficial Effects of Vitamin D Treatment in an Obese Mouse Model of Non-Alcoholic Steatohepatitis
Source: Nutrients. 2019 Jan 3;11(1):77. doi: 10.3390/nu11010077 (PMC6356425; doi:10.3390/nu11010077)
Supplement: Supplementary file 1 [file nutrients-11-00077-s001.pdf]

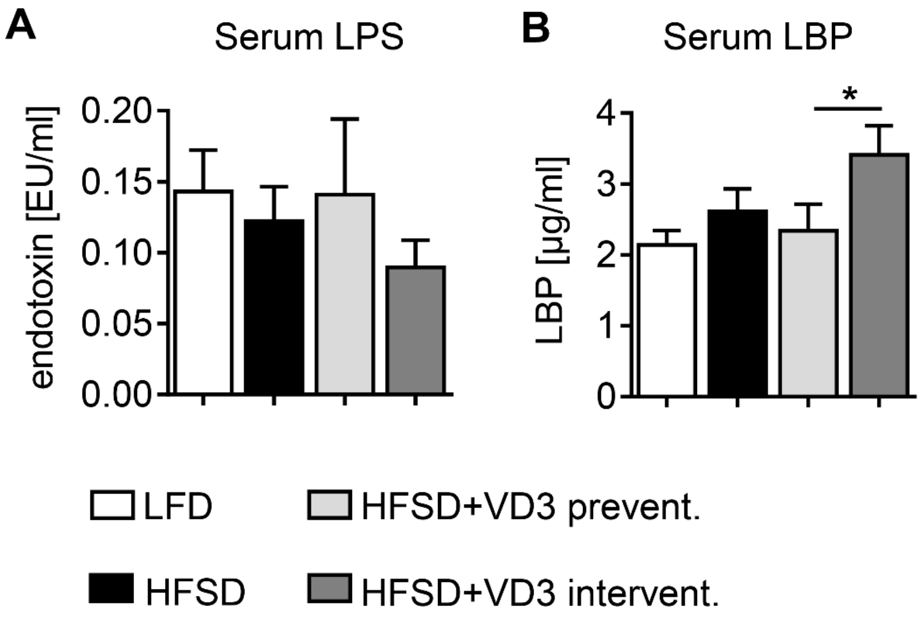

Figure S1. Effects of preventive and interventional vitamin D treatment on serum levels of lipopolysaccharide (LPS) and LPS-binding protein (LBP). Levels of (**A**) serum LPS and (**B**) serum LBP were measured as outlined in the Material and Methods section. Data represent mean +/- SEM derived from n = 9 for LFD and n = 10 for all the other groups.
